# The Parisian Confrère as Deputy Physician

**Published:** 1859-06

**Authors:** 


*The Parisian Confrère as Deputy Physician.*—"To go away and trust your patients to a *confrère*, is to furnish a dangerous opportunity to one of the crying appetites of the day. I rather would shut my door, and tell the porter to say I was absent, than recommend my patients to a confrère. If the case is not urgent, they will wait till I return; if they will not wait, hazard perhaps will serve them worse than I should by handing them over to a *confrère*, whom they will appreciate less than they do me. The chosen *confrère* who supplies my place is sure to make himself very amiable, very attentive, *empressé* and *prévenant*, out of esteem and affection for me. He will discover that he has a conquest to make, and he will use his best means to gain it. An illustrious doctor, who lived at the beginning of this century, was wiser. He lived in grand style, and every year took two good months' vacation. And what sort of a man did he leave as his substitute? He himself was an 'elegant' man of the world, spiritual, lettered, catholic, or pretending to be so; all his clients were people of the court, noble dames, and bishops; and he chose for his substitute a very worthy *confrère*, to whom all the world did justice, but one who was timid, reserved, careless of dress, abrupt in his address, and but little of a Voltairien. The contrast was striking, and we may guess to his profit."—*L' Union Médicale.*

